# Correlates of sexual function in women with acute lumbar disc herniation in Iran: a cross-sectional study

**DOI:** 10.1038/s41598-024-57274-w

**Published:** 2024-03-18

**Authors:** Zahra Moradi, Shadab Shahali, Fazlollah Ahmadi, Ali Montazeri

**Affiliations:** 1 Department of Reproductive Health and Midwifery, Faculty of Medical Sciences, Tarbiat Modares Univesity, Tehran, Iran; 2 Department of Nursing, Faculty of Medical Sciences, Tarbiat Modares University, Tehran, Iran; 3 Health Metrics Research Center, Institute for Health Sciences Research, ACECR, Tehran, Iran; 4 Faculty of Humanity Sciences, University of Science & Culture, Tehran, Iran

**Keywords:** Intervertebral disk herniation, Lumbar vertebrae, Sexual activity, Women, Low back pains, Quality of life, Health care

## Abstract

Evidence suggests that lumbar disc herniation** (**LDH) influences sexual function to a great deal. However, most existing studies have been conducted on men. Thus, the current study aimed to assess sexual function and its correlates in women with acute LDH. This descriptive cross-sectional study was conducted on 350 women of reproductive age with acute LDH in Fasa, Iran in 2023. The mean score of female sexual function was 21.33 (3.38). Almost 80% of women had sexual dysfunction. Women scored lower on sexual desire and the higher on lubrication. As the severity of LDH increased, arousal, lubrication and sexual pain score decreased and low back pain (LBP) score increased (*p* < 0.05). The number of sexual activities after disc herniation was significantly lower in the women with sexual dysfunction (*p* < 0.001). Regression analysis showed a significant association between sexual dysfunction and LBP intensity (OR = 1.13, CI 1.02–1.26, *p* = 0.01) and lumbar disc herniation intensity (OR = 2.22, CI 1.07–4.62, *p* = 0.03). Lumbar disc herniation (LDH) may significantly affect women's sexual function. Severity of low back pain and severity of lumbar disc herniation were found to be risk factors for sexual dysfunction in this population.

## Introduction

The lumbar spine, located in the lower back, consists of five vertebrae and intervertebral discs between them. Lumbar disc herniation (LDH) occurs when the outer layer of one of these discs sobbing, causing the soft, gel-like material inside to bulge or leak out. This may cause irritation or compression of the nearby nerve, leading to pain and other symptoms^1^. The most common symptoms of LDH include lower back pain (LBP), leg pain, and numbness or weakness in the affected area. This condition can significantly impact a person's physical function and daily life. The pain and discomfort associated with LDH can make it difficult to bend forward, twist, or move the back in certain ways. Individuals may also experience muscle spasms that further limit their range of motion. LDH can cause muscle weakness or atrophy due to the compression or irritation of nerves that control muscle function. This can lead to difficulty standing, walking, or performing simple tasks such as lifting objects^2,3^ LDH commonly affects sexual function, a key indicator of quality of life^3^. LDH can impact sexual activity through LBP, leg pain, nerve compression, and side effects of pain medication^4,5^. LDH has an incidence of about 5–20 cases per 1000 adults per year and typically occurs in individuals in their third to fifth decade of life, a time when couples are usually sexually active^6^. Sexual health is discussed significantly less often with female patients than with male patients, leading to most studies on the correlation between sexual function and LDH being conducted on men^7^. This could be attributed to social norms and expectations surrounding sexual performance, making it easier for physicians to address men, despite data showing that sexual dysfunction is more common in women^1,8^. Research on the sexual function of women with acute LDH is very limited. Akkurt et al. examined the impact of Lumbar disc disease (LDD) on female sexuality. They found that patients with lumbar disc disease had lower Female Sexual Function Index (FSFI) scores across all subgroups^9^. Most studies compared sexual function before and after herniated disc surgery, with the prevalence of sexual dysfunction ranging from 26.6 to 80%^1,3,4,9–15^. Long-term follow-up indicated poor recovery after surgery^16^.

Given that this condition commonly affects reproductive-aged women than men^1,8^ and since most studies on the correlation between sexual function and LDH being conducted on men^7^, our study aimed to examine the sexual function of women with acute LDH and the factors contributing to sexual dysfunction in this population. We looked at correlations between LDH and different aspects of sexual function, such as desire, arousal, lubrication, orgasm, satisfaction, and pain, to identify the most affected areas due to disc herniation.

## Methods

### Study design and setting

This was a cross-sectional study that was conducted in the health centers affiliated to Fasa University of Medical Sciences, Fasa, Iran, in 2023. The project was approved by the Ethics Committee of Tarbiat Modares University, Tehran, Iran (Code of Ethics: IR.MODARES.REC.1401.168).

### Participants

A sample of women of reproductive age with acute lumbar disc herniation (LDH) detected by magnetic resonance imaging and confirmed by a neurosurgeon or orthopedist were recruited. As such the main investigator (ZM) attended the selected healthcare centers and the magnetic resonance imaging (MRI) centers in Fasa, and provided a list of women of reproductive age who had MRI for the last year. Then the researcher contacted women, provided them with the necessary explanations, and asked them to take part in the project. Women who met the inclusion criteria, were entered into the study.

### Inclusion and exclusion criteria

Inclusion criteria were willingness to participate in the study, Iranian nationality, ability to read and write, age between 18 and 55 years, being married and having sex in the last six months, no underlying diseases affecting sexual function according to self-report, not taking any medications affecting sexual function, not having husband's impotence, and not being pregnant or lactating at the time of the study. Women were excluded if they failed to complete the study questionnaires. All women signed the informed consent form.

### Sample size

The sample size was determined based on the following formula and according to the prevalence of sexual dysfunction in women with LDH (66%)^12,16^. Considering the study with 80% power at 5% significance level and precision of 5% a sample size of 350 women was estimated.

$$ {\text{Z}}^{2} {\text{P}}\left( {{1} - {\text{P}}} \right)/{\text{d2}}\;{\text{where}}\;{\text{Z}} = {1}.{96},\;{\text{d}} = \% {5},\;{\text{and}}\;{\text{P}} = \% {66} $$

### Measures

The following questionnaires were used to collect the data:

1. The demographic, obstetric and LDH characteristics questionnaire containing items related to demographic characteristics such as age, level of education, employment status, income, as well as obstetric records such as marriage duration, gravidity, parity, number of abortions, and items related to LDH characteristics such as duration of lumbar disc herniation, location of disc herniation, and disc herniation intensity.
2. The Visual Analogue Scale (VAS) to assess the low back pain intensity. This is a 10-point scale where 0 indicates no pain and 10 indicates the highest level of pain^17^. In the last one month.
3. The Female Sexual Function Index (FSFI): Sexual function was assessed by the FSFI. It is a brief, 19-item, self-report, multi-dimensional questionnaire. The questionnaire contains 6 sub-scales namely: sexual desire, sexual arousal, lubrication, orgasm, satisfaction and pain. It uses a 5-point Likert scale ranging from 1 to 5 with higher scores indicating greater levels of sexual functioning. The scoring could be calculated for both the total and the subscales^18^. The FSFI Desire domain contains 2 items that scores range from 1.2 to 6. Arousal domain contains 4 items and scores range from 0 to 6. Four items form the subgroup of lubrication domain scores range from 0 to 6. The Orgasm domain contains 2 items. This domain scores range from 0 to 6. The Satisfaction domain includes 3 items. Scores on this domain range from 0.8 to 6. The Pain domain contains 3 items, and domain scores range from 0 to 6^19^. Since the number of questions in the subgroups are not equal to each other, first we added the scores from the questions of each subgroup to equalize the weighting of the subgroups with each other. (0.6 for desire; 0.3 for arousal; 0.3 for lubrication; 0.4 for orgasm; 0.4 for satisfaction; and 0.4 for pain) The total score of the scale is obtained by adding the scores of the six subgroups. The maximum score for each domain will be 6 and for the whole scale will be 36. A score of zero means that the person has not had sexual activity during the last 4 weeks^18,20^. The cut-off point for the total scale and subscales are as follows: total scale 28, desire 3.3, arousal 3.4, lubrication 3.4, orgasm 3.4, satisfaction 3.8, and sexual pain 3.8. The validity and reliability of the Persian version of the FSFI were shown in a study. The Cronbach’s alpha for the six FSFI domains was: desire 0.72, arousal 0.90, lubrication 0.90, orgasm 0.91, satisfaction 0.76, pain 0.88 and the total scale 0.92^21^.

### Statistical analysis

Data were analyzed using SPSS software version 26. Descriptive statistics such as frequency, percentage, mean and standard deviation were used to explore the data. Independent t-tests, one-way analysis of variance (ANOVA), and chi-squared were used to compare all independent variables study between two groups with and without sexual dysfunction. Also, to examine the relationship between dependent variable (sexual function) and independent variables including demographic, obstetric and disc herniation characteristics both univariate and multivariable logistic regression analyses were performed and odds ratio and 95% confidence intervals are reported. All variables were entered separately in the univariate regression model.

### Ethics approval and consent to participate

The project was approved by the Ethics Committee of Tarbiat Modares University, Tehran, Iran (Code of Ethics: IR.MODARES.REC.1401.168). All research was performed in accordance with the Declaration of Helsinki. Informed consent was obtained from all participants after a thorough explanation of the study objectives. Confidentiality and voluntary participation were assured and participants had all rights to withdraw at any time.

## Results

### Participants

Three Hundred and seventy questionnaires were distributed, of which 12 were excluded from the study due to Incomplete filling of the demographic information and 8 were excluded because they didn't complete the sexual function questionnaire.

The mean (SD) age of participant was 40.79 (6.56) years ranging from 22 to 55. The mean (SD) marriage duration was 19.04 (8.51) years ranging from 1 to 44. The most common location of disc herniation was between L4-L5, followed by L5-S1, and the highest number of multi-vertebral injuries was between L4-L5-S1 vertebrae. A majority of patients (78.3%) had only one-disc herniation, and 21.7% had more than one disc involved. In terms of the intensity of the disc, the mean intensity has the largest percentage. Most patients had lower lumbar disc herniation (LLDH) (72.9%). In all 95 cases had a history of surgery. Of these 12 cases had more than one operation for spinal disc herniation, and the highest number of surgeries was 4, and two women had a history of 4 times discectomy surgery. The minimum time since the surgery was 1 year and the maximum time was 13 years. The mean (SD) number of sexual activities per month after having a herniated disc was 5.91(4.05).

### Female sexual function

The mean score (SD) for the female sexual function index was 21.33 ± 3.38), ranging from 2 to 34.5. Using the cut-off point of 28, 284 women (80.1%) were identified as experiencing sexual dysfunction. The highest mean (SD) score was for satisfaction (4.33 ± 1.45), and the lowest mean (SD) score was for desire (2.84 ± 1.01). The mean score of desire (2.84 ± 1.01), arousal (3.14 ± 1.64), pain (3.5 ± 1.84), and total score of sexual function (21.33 ± 3.38) were lower than the cut-off points.

### Comparison of independent variables of the study in women with and without sexual dysfunction

The mean score for sexual function in all subgroups of women with sexual dysfunction was notably lower than in women without sexual dysfunction (*p* < 0.001).

Significant correlations were observed between sexual dysfunction and education level (*p* = 0.007), income (*p* = 0.01), disc herniation intensity (*p* < 0.001), as well as age (*p* < 0.001), duration of marriage (*p* < 0.001), number of living children (*p* = 0.004), gravidity (*p* = 0.004), parity (*p* = 0.01), interval from last delivery (*p* < 0.001), and back pain intensity score in the last month (*p* < 0.001). Additionally, the number of sexual activities in the month after disc herniation was notably lower in the dysfunctional group (*p* < 0.001) (Table 1).

**Table 1:** Comparison of Socio-demographic, obstetrics and LDH variables in women with and without sexual dysfunction (n = 350).

	Total (n = 350)	With sexual dysfunction (n = 284)	Without sexual dysfunction (n = 66)	p*
	Number (%)	Number (%)	Number (%)	
Education				0.02
Primary	61 (17.4)	57 (93.4)	4 (6.6)	
Secondary	153 (43.7)	122 (79.7)	31 (20.3)	
Higher	136 (38.9)	105 (77.2)	31 (22.8)	
Employment				0.13
Housewife	209 (59.7)	175 (83.7)	34(16.3)	
Employed	141 (40.3)	109 (77.3)	32(22.7)	
Income				0.01
Poor	88 (25.1)	80 (90.9)	8 (9.1)	
Intermediate	162 (46.3)	131 (80.4)	32 (19.6)	
Good	100 (28.6)	73 (73.7)	26 (26.3)	
Housing situation				0.13
Personal	266 (76)	220 (83)	45 (17)	
Renting	84 (24)	64 (75.3)	21 (24.7)	
BMI				0.35
≤ 18.5	9 (2.6)	7 (77.8)	2 (22.2)	
18.5 < BMI ≤ 24.9	97 (27.7)	73 (75.3)	24 (24.7)	
25 < BMI ≤ 29.9	161 (46)	135 (83.9)	26 (16.1)	
BMI ≥ 30	83 (23.7)	69 (83.1)	14 (16.9)	
Last delivery type				0.65
NVD	133 (38)	109 (82)	24 (18)	
Cesarean section	194 (55)	158 (81.4)	36 (18.6)	
No delivery	23 (7)	17 (73.91)	6 (26.09)	
Abortion				0.68
Yes	135 (38.6)	111 (82.8)	24 (17.8)	
No	215 (61.4)	173 (80.5)	42 (19.5)	
Discectomy surgery				0.55
Yes	95 (27.1)	79 (83.2)	16 (16.8)	
No	255 (72.9)	205 (80.4)	50 (19.6)	
LDHI				0.007
Mild	117 (33.4)	87 (74.4)	30 (25.6)	
Moderate	140 (40)	112 (80)	28 (20)	
Severe	93 (26.6)	85 (91.4)	8 (8.6)	
Place of disc herniation				0.91
ULDH	59 (16.9)	49 (83.1)	10 (16.9)	
LLDH	255 (72.9)	206 (80.8)	49 (19.2)	
ULLDH	36 (10.2)	29 (80.6)	7 (19.4)	
Number of disc herniation				0.44
One disc	274 (78.28)	220 (80.3)	54 (19.7)	
More than one disc	76 (21.72)	64 (84.2)	12 (15.8)	
Age (years)	40.79 (6.56)	41.33(6.55)	38.45 (6.09)	< 0.001
Marriage duration (year)	19.04 (8.51)	19.85 (8.39)	15.56 (8.18)	< 0.001
Gravidity	2.76 (1.53)	10.83 (7.38)	8 (6.75)	0.005
Parity	2.12 (1.04)	2.18 (1.04)	1.87 (1.04)	0.03
Number of living children	2.11 (1.01)	2.17 (1.03)	1.84 (0.97)	0.01
Interval from last delivery (year)	10.30 (7.34)	18.85 (8.39)	15.56 (8.18)	< 0.001
Number of abortions	1.04 (0.63)	0.63 (1.06)	0.62 (0.97)	0.93
LDHD (year)	7.26 (5.28)	7.25 (5.18)	7.31 (5.76)	0.9
LBPI in the last month (0–10)	6.30 (2.54)	6.53 (2.45)	5.30 (2.71)	< 0.001
Distance to the time of the last discectomy surgery (year)	0.96 (2.18)	1 (1.20)	0.81 (2.09)	0.53
Number of discectomy surgeries	0.32 (0.59)	0.32 (0.61)	0.3 (0.58)	0.79
The number of sexual activities in a month after having a herniated disc	5.91 (4.05)	5.43 (40.6)	7.95 (3.35)	< 0.001

LDH, Lumbar disc herniation; NVD, Normal vaginal delivery; LDHI, Lumbar disc herniation intensity; ULDH, Upper lumbar disc herniation; LLDH, lower lumbar disc herniation; ULLDH, Upper lower lumbar disc herniation; LBPI, Low back pain intensity; LDHD, Lumbar disc herniation Duration.

*Chi-square, **t-test, SD, Standard deviation.

### Comparison of sexual function and low back pain intensity in LDH groups

Women who underwent discectomy surgery had notably lower lubrication (*p* = 0.02) and orgasm (*p* = 0.01) scores. Those with mild disc herniation showed higher arousal (*p* = 0.03) and lubrication (*p* = 0.04) scores compared to those with severe disc herniation. Additionally, women with mild and moderate disc herniation reported less sexual pain than those with severe disc herniation (*p* = 0.004), and those with mild disc herniation experienced less back pain compared to women with moderate and severe disc herniation (*p* < 0.001). There was no significant relationship between disc herniation location, back pain, and the score of subgroups of the sexual function (*p* > 0.05). Women with only one disc herniation had notably higher desire (*p* = 0.01), arousal (*p* = 0.03), and orgasm (*p* = 0.02) scores compared to women with multiple disc herniation, as well as less sexual pain (*p* = 0.01) (Table 2).

**Table 2:** Comparison of mean scores of the six domains of the FSFI and LBPI in different groups of LDH.

	Desire mean (SD)	*p*	Arousal mean (SD)	*p*	Lubrication mean (SD)	*p*	Orgasm mean (SD)	*p*	Satisfaction mean (SD)	*p*	Pain mean (SD)	*p*	LBPI mean (SD)	*p*
Discectomy history		0.3*		0.1*		0.01*		0.01*		0.17*		0.1*		0.16*
Yes	2.75(1.12)		2.91(1.76)		3.39(2.04)		3.33(1.99)		4.16(1.60)		3.23(1.98)		5.98(2.79)	
No	2.87(0.97)		3.23(1.59)		3.92(1.74)		3.86(1.81)		4.40(1.39)		3.60(1.78)		6.41(2.47)	
LDHI		0.2**		0.03**		0.04**		0.07**		0.12**		0.004**		< 0.001**
Mild	2.95(1.05)		3.45(1.63)		4.03(1.75)		4.01(1.85)		4.55(1.42)		3.78(1.86)		5.29(2.95)	
Moderate	2.81(0.99)		3.06(1.64)		3.82(1.83)		3.66(1.79)		4.25(1.5)		3.62(1.79)		6.77(1.97)	
Severe	2.73(1)		2.87(1.62)		3.39(1.9)		3.42(1.85)		4.18(1.39)		2.96(1.79)		6.87(2.40)	
Place of LDH		0.47**		0.75**		0.56**		0.99**		0.82**		0.24**		0.94**
ULDH	2.81(1.05)		3.27(1.55)		4.01(1.72)		3.74(1.82)		4.26(1.51)		3.82(1.87)		6.30(2.82)	
LLDH	2.87(1)		3.1(1.64)		3.73(1.85)		3.71(1.88)		4.33(1.44)		3.47(1.82)		6.32(2.48)	
ULLDH	2.65(1.06)		3.21(1.87)		3.76(1.97)		3.71(1.99)		4.45(1.43)		3.20(1.86)		6.16(2.55)	
Number of LDH		0.01*		0.03*		0.11*		0.02*		0.09*		0.01*		0.17*
One disc	2.91(1)		3.24(1.58)		3.76(1.75)		3.83(1.79)		4.40(1.44)		3.63(1.76)		6.20(2.53)	
More than one disc	2.51(1)		2.78(1.81)		3.48(2.10)		3.29(2.10)		4.08(1.48)		3.03(2.04)		6.65(2.58)	

FSFI, Female sexual function index; LDH, Lumbar disc herniation; LDHI, Lumbar disc herniation Intensity; LBPI, Low back pain Intensity; ULDH, Upper lumbar disc herniation; LLDH, Lower lumbar disc herniation; ULLDH, Upper and lower lumbar disc herniation together; SD, Standard Deviation.

*t-test, ** one-way ANOVA.

The duration of lumbar disc herniation did not affect sexual dysfunction in subgroups (*p* > 0.05). The back pain score in women who had dysfunction in the subgroups was significantly higher than women who did not have dysfunction (*p* < 0.05). The number of monthly sexual activities of women who had dysfunction in subgroups was significantly less than women who did not have dysfunction (*p* < 0.001) (Table 3).

**Table 3:** Comparison of mean scores of quantitative values of LDH factor in subgroups of sexual dysfunction.

	LDHD mean (SD)	p*	LBPI mean (SD)	p*	The number of sexual activities in a month after having a herniated disc mean (SD)	p*
Desire disorder		0.65		0.004		< 0.001
Yes	7.36(5.35)		6.62(2.44)		4.93(4.08)	
No	7.10(5.19)		5.82(2.63)		7.36(3.56)	
Arousal disorder		0.43		0.002		< 0.001
Yes	7.48(5.18)		6.73(2.44)		4.48(4.23)	
No	7.04(5.39)		5.88(2.58)		7.28(3.36)	
Lubrication disorder		0.76		0.001		< 0.001
Yes	7.13(4.69)		6.99(2.52)		3.91(4.01)	
No	7.31(5.53)		6.00(2.50)		6.77(3.76)	
Orgasmic disorder		0.21		0.03		< 0.001
Yes	7.78(4.92)		6.73(2.47)		4.19(4.34)	
No	7.02(5.43)		6.10(2.56)		6.68(3.67)	
Satisfaction disorder		0.10		0.008		< 0.001
Yes	7.93(5.51)		6.83(2.43)		3.99(3.92)	
No	6.94(5.16)		6.05(2.56)		6.81(3.80)	
Pain disorder		0.23		< 0.001		< 0.001
Yes	6.91(4.68)		6.82(2.38)		4.98(4.30)	
No	7.58(5.78)		5.81(2.61)		6.77(3.61)	

LDH, Lumbar disc herniation; LDHD, Lumbar disc herniation Duration (year); LBPI, Low back pain Intensity; SD, Standard deviation.

*t-test.

### Association between sexual function and LDH

All variables were entered separately in the univariate regression model. Increasing the level of education, income, and the number of sexual activities in the month after disc herniation decreased the probability of sexual dysfunction. Conversely, increasing the severity of disc herniation, age, marriage duration, gravidity, parity, number of living children, interval from last delivery, and back pain severity score in the last month increased the probability of sexual dysfunction. Subsequently, using multiple regression analysis, the simultaneous effect of variables with *p* ≤ 0.20 was examined using the enter method. According to the final model, among those with severe lumbar disc herniation, the probability of sexual dysfunction was 2.22 higher as compared to those with mild lumbar disc herniation intensity (OR = 2.22, CI 1.07–4.62, *p* = 0.03), and with one unit increase in the low back pain intensity, the probability of sexual dysfunction increased by 13% (OR = 1.13, CI 1.02–1.26, *p* = 0.01) (Table 4).

**Table 4:** Association between sexual dysfunction and LDH according to multiple logistic regression analysis (n = 350).

	AOR	95% CI	*p*
Education			
Primary	1.00 (ref.)		
Secondary	0.76	0.28–2.06	0.59
Higher	1.60	0.46–5.54	0.45
Employment			
Housewife	1.00 (ref.)		
Employed	0.70	0.37–1.35	0.29
Housing situation			
Renting	1.00	1.00	
Personal	1.34	0.74–2.42	0.32
Income			
Poor	1.00 (ref.)		
Intermediate	0.68	0.30–1.50	0.34
Good	0.44	0.17–1.09	0.07
LDHI			
Mild	1.00 (ref.)		
Moderate	1.58	0.87–2.87	0.13
Severe	2.22	1.07–4.62	0.03
Age (years)	1.02	0.95–1.8	0.5
Marriage duration (year)	1.03	0.96–1.11	0.27
Gravidity	1.24	0.93–1.65	0.13
Parity	0.32	0.1–1.04	0.52
Number of living children	2.10	0.65–6.74	0.2
Interval from last delivery (year)	1.05	0.98–1.11	0.13
LBPI in the last month (0–10)	1.13	1.02–1.26	0.01

LDH, Lumbar disc herniation; LDHI, Lumbar disc herniation intensity; LBPI, Low back pain intensity; AOR, Adjusted odds ratio; CI, Confidence interval.

## Discussion

Our data revealed that 80.1% of women with LDH had sexual dysfunction. Women with spine pathology are more likely to experience sexual dysfunction than men, with a ratio of 10:6^8^. Reports on sexual dysfunction and lumbar disc herniation (LDH) are predominantly focused on men, which is attributed to challenges in discussing sexual performance with female patients^22,23^. As no specific study on the sexual performance of women with LDH was found, statistics related to men's studies or combined results of men and women were included for discussion. The prevalence of sexual dysfunction in women with LDH varies across studies. It has been reported between different studies from 53.8 to 100%^12–14,23–28^; this variance may be influenced by cultural and social factors, as well as the use of different questionnaires to measure sexual function. Kumar's study indicated that only men reported sexual dysfunction, possibly due to cultural factors and feelings of shame in women^16^. Pronin demonstrated that the registration rate of sexual dysfunction in patients with cauda equina syndrome, one of the most serious and complex spinal pathologies, was much lower (6.7%) than other symptoms of the disease, and even educating physicians about the importance of recording symptoms did not increase this rate^25^. Therefore, it appears that these few studies of sexual function in women with LDH may be underreporting the true extent of sexual dysfunction in these women. However, all studies reported a prevalence of over 50%, underscoring the importance of the problem in these women and warranting increased attention from health care professionals.

Women without sexual dysfunction exhibited higher levels of education and incomes, both of which are indicative of a higher quality of life. This could potentially impact their sexual function, a key component of overall well-being. A higher level of education can have a positive effect on the quality of a woman's sexual life. According to the World Health Organization (WHO), maintaining sexual activity is a marker of good health^28^. Education can provide individuals with a better understanding of their bodies, sexual health, and relationships, leading to improved sexual experiences. Higher levels of literacy among women are associated with health literacy. Research has shown that health literacy, which includes factors such as access to information, reading, understanding, evaluation, and decision-making, can positively affect sexual function and satisfaction. Studies have shown a significant relationship between health literacy, education, and occupation with sexual function in women. In addition, sexual health literacy has been associated with increased sexual satisfaction and quality of life in women^29^. Higher income levels may lead to better sexual performance in women through several mechanisms. Higher income levels can provide women with greater financial independence and control over their lives, which can lead to improved self-esteem and confidence in their sexual relationships. In addition, higher income levels can provide women with access to better health care and education, which can improve their overall health and well-being, including their sexual health^30^.

Conversely, women with sexual dysfunction tended to be older and to have had more pregnancies and births. They were also more likely to have been married for a longer period of time. As women age, there may be physiological changes that affect sexual desire and function, such as hormonal fluctuations and physical changes. A combination of physical, psychological, and relational factors associated with aging, pregnancy, and long-term relationships may contribute to decreased sexual performance in women. Sangondimath demonstrated that sexual dysfunction tends to increase with age in women^26^. Furthermore, for men with LDH, being over 40 years old and having a marriage duration of more than 10 years were linked to reduced scores in ejaculation, sexual satisfaction, and overall sexual function^1^.

The current study revealed that more severe disc herniation correlated with increased back and sexual pain and sexual dysfunction. The findings suggest that sexual pain may stem from back pain caused by disc herniation. Women experiencing more severe back pain engaged in less frequent monthly sexual activity. Women with sexual dysfunction had higher back pain scores compared to those without sexual dysfunction across 6 subgroups of sexual function, and reported lower monthly sexual activity. Lumbar disc herniation can negatively affect sexual life, leading to decreased sexual desire and decreased sexual intercourse. The pain and discomfort associated with lumbar disc herniation can reduce willingness to engage in sexual activity, which can further exacerbate sexual dysfunction. Additionally, chronic low back pain can lead to emotional distress and anxiety, which can also impact sexual function. Acka stated that chronic back pain and depression are the primary causes of sexual dysfunction^31^.

Saritas demonstrated that in patients with LDH, the effects of nerve pressure, pain, and psychological factors can significantly impact their quality of life and sexual performance^5^. A study found that patients with LDH had a 78% decrease in sexual activity, and 64% of them were hesitant to engage in sex^24^. 54% of men and 76% of women with LDH needed to adjust their position during sexual intercourse^8^.

In our research, women with LDH displayed varying degrees of disorder across different subgroups, with desire, arousal, pain, orgasm, satisfaction, and lubrication being the most to least affected, respectively. Other studies have indicated that LDH may lead to reduced libido, erectile dysfunction, arousal disorders, and orgasmic disorders, potentially accompanied by pain and nerve compression^5^. LDH has been found to have adverse effects on sexual life, with decreased libido and reduced sexual activity being commonly reported issues^24^. Complications of LDH, such as neurological disorders, severe pain, and limitations in physical activity, can contribute to a decline in quality of life, leading to depression, and ultimately impacting not only individuals' social lives but also their sexual lives and relationships with their partners. Chronic diseases can cause pain, resulting in decreased sexual desire and satisfaction, as well as affecting the frequency and duration of sexual intercourse^9^. After experiencing LDH, 54% of men reported a decrease in sexual activity, and 77.3% reported a decrease in sexual desire and satisfaction^1^. The most prevalent disorder resulting from LDH in women is orgasmic disorder^24^. Female patients commonly exhibited dysfunction in libido, maintenance of arousal, and orgasm disorder, with back pain being the most frequently reported physical symptom for which patients sought medical assistance^32^.

Most studies, including our own, have shown that the area most affected is libido and orgasm. Overall, lumbar disc herniation can have a significant impact on various aspects of a woman's sexual performance, leading to decreased sexual satisfaction and overall well-being. However, the sexual response cycle can be disrupted in various ways, ultimately leading to sexual dysfunction and a decrease in sexual activity frequency. It is crucial to recognize that the primary cause of sexual dysfunction in these women is back pain and mental issues stemming from disc herniation^31^. Therefore, in order to address sexual dysfunction, we must address the complications caused by LDH. Lack of sexual education, shyness, and secrecy can make it challenging to seek help. Educating patients about the nature of LDH, surgical intervention, and post-operative care can increase awareness and impact sexual satisfaction^5^.

The location of the herniated disc in the spine, whether upper lumbar or lower lumbar, can have different effects on women's sexual function. Our study found that the location of disc herniation did not impact sexual function. However, herniation of more than one disc led to a decrease in desire, arousal, and orgasm scores, as well as an increase in sexual pain. The duration of disc herniation, on the other hand, did not affect women's sexual performance. Overall, the presence of multiple lumbar disc herniations may exacerbate sexual difficulties in women, emphasizing the importance of addressing and managing sexual problems in patients with this condition. This means that LDH intensity and the number of discs involved are more influential than the disc location.

Additionally, our study revealed that low back pain intensity and lumbar dick herniation intensity were factors affecting sexual dysfunction in women with LDH. Severe lumbar disk herniation raised the chance of sexual dysfunction by 22%, and with each rise in low back pain intensity score, the likelihood of sexual dysfunction increased by 13%. As described in the Anxiety Pain Cycle^33^, back pain causes pain during intercourse, and in some positions, such as the missionary position, the back pain increases. As the back pain increases, so does the sexual pain. Gradually, the pain reduces the woman's libido, the decrease in female sexual desire leads to a decrease in the frequency of sexual interactions, ultimately both of these lead to sexual dysfunction.

### Limitations and strengths

One of the strengths of this study compared to similar studies is that it has a large sample size and examined LDH variables such as severity, location, surgical history, and duration. Also, unlike other previous studies, it is not single-center. We can mention the following as limitations of our study: The reliance on self-reported measures introduces the potential for bias and recall inaccuracy, which may affect the validity of the findings. A cross-sectional design prevents the establishment of causal relationships and temporal sequences. If the husbands of these women were also included in the study, perhaps more interesting results would be obtained. Since we did not find a study similar to ours, it was not possible to compare some of the results.

## Conclusion

In conclusion, Lumbar disc herniation (LDH) can significantly influence women’s sexual function. By reducing back pain, it is also possible to reduce sexual pain and sexual dysfunction. Women can improve their sexual performance by maintaining sexually active. It is important to seek medical attention and follow recommended treatment plans to manage this condition effectively.

## Data Availability

The data sets used and/or analyzed during the current study available from the corresponding author on reasonable request.
